# Individualized lung recruitment during high-frequency ventilation in preterm infants is not associated with lung hyperinflation and air leaks

**DOI:** 10.1007/s00431-016-2744-4

**Published:** 2016-06-21

**Authors:** Anne P. De Jaegere, Eline E. Deurloo, Rick R. van Rijn, Martin Offringa, Anton H. van Kaam

**Affiliations:** Department of Neonatology, Emma Children’s Hospital, Academic Medical Center, Amsterdam, The Netherlands; Department of Radiology, Academic Medical Center, Amsterdam, The Netherlands; Department of Pediatric Clinical Epidemiology, Emma Children’s Hospital, Academic Medical Center, Amsterdam, The Netherlands

**Keywords:** Neonatal respiratory distress syndrome, Pneumothorax, Thoracic radiograph, Diagnosis

## Abstract

Lung recruitment during high-frequency ventilation (HFV) in preterm infants with respiratory distress syndrome (RDS) has been associated with an increased risk of lung hyperinflation and air leaks. Individualizing the lung recruitment procedure to the severity of lung disease of each patient might reduce these risks. In this prospective cohort study, we evaluated chest X-ray (CXR) characteristics during individualized oxygenation-guided lung recruitment with HFV in preterm infants with RDS, before and after surfactant therapy. Two pediatric radiologists scored radiolucency, the presence of lung hyperinflation, and/or air leaks following lung recruitment during HFV in 69 infants before and 39 infants after surfactant treatment. Following lung recruitment, the median radiolucency score was 2, with 44 (64 %) infants having a score ≤2. Only mild to moderate hyperinflation was seen in 13 (19 %) infants, with no air leaks. After the surfactant, the radiolucency score improved in 62 % of 39 paired CXRs (*p* < 0.001). Mild to moderate hyperinflation was seen in nine (24 %) patients. During the entire admission, only four (6 %) of the patients developed air leaks.

*Conclusion*: The risk of significant hyperinflation and air leaks is low when using an individualized oxygenation-guided recruitment procedure during HFV in preterm infants with RDS.

**Table:** 

**What is Known:** • *Lung recruitment during high-frequency ventilation in preterm infants with respiratory distress syndrome is associated with an increased risk of lung hyperinflation and air leaks.*
**What is New:** • *The risk of lung hyperinflation and air leaks is low when using an individualized oxygenation-guided lung recruitment procedure during high-frequency ventilation in preterm infants with respiratory distress syndrome.*

## Introduction

Respiratory distress syndrome (RDS) is one of the most common causes of respiratory failure in preterm infants. Despite the increasing use of primary nasal continuous positive airway pressure (nCPAP), many infants with RDS still need to be intubated, mechanically ventilated, and treated with exogenous surfactant to restore lung function and gas exchange [1]. However, mechanical ventilation can also injure the immature lung and is considered an important risk factor for developing bronchopulmonary dysplasia (BPD) [9].

High-frequency ventilation (HFV) is a frequently used lung-protective ventilation mode in preterm infants with RDS [18, 22]. Animal and human studies have shown that HFV is only lung protective if combined with a recruitment procedure designed to reverse atelectasis and stabilize lung volume at functional residual capacity, i.e., the optimal lung volume or open-lung ventilation strategy [5, 12]. However, there have been concerns that lung recruitment during HFV may increase the risk of lung hyperinflation and air leaks in infants with RDS [3, 4, 19].

We and others have emphasized the importance of individualizing the recruitment procedure during HFV using oxygenation as an indirect bedside tool to optimize lung volume [6, 15]. Taking advantage of the lung hysteresis that is present in most infants with RDS [13], lung volume is stepwise recruited and stabilized with a continuous distending pressure (CDP) tailored to the severity of RDS in each individual patient. To date, the effect of such an individualized oxygenation-guided recruitment procedure on the risk of lung hyperinflation and air leaks during HFV has not been studied.

Therefore, the aim of this prospective cohort study is to evaluate the changes in chest X-ray (CXR) characteristics of preterm infants with RDS ventilated with primary individualized open-lung HFV. We were especially interested in (1) the changes in lung aeration after lung volume optimization and (2) the incidence of hyperinflation and air leak syndrome.

## Materials and methods

### Patients and ventilation protocol

The prospective cohort study was performed in the neonatal intensive care unit of the Emma Children’s Hospital, Academic Medical Center (Amsterdam, the Netherlands). The institutional review board approved the study, and the study was in accordance with the ethical standards laid down in the 1964 Declaration of Helsinki and its later amendments.

In our unit, preterm infants (gestational age <37 weeks) with a suspected diagnosis of RDS and failing nCPAP are treated with primary open-lung HFV using either a SensorMedics 3100A oscillator (CareFusion, Yorba Linda, CA) or a Babylog 8000 plus (Drager Medical, Lubeck, Germany). A detailed description of this open-lung procedure has been previously described [6]. Briefly, HFV is started at a continuous distending pressure (CDP) of 6–8 cm H_2_O, a pressure amplitude resulting in a slight wiggle of the chest, a frequency of 10 Hz, and an inspiration time of 33 % (SensorMedics 3100A only). Collapsed alveoli/sacculi are first recruited by stepwise increasing of the CDP, until the fraction of inspired oxygen (FIO_2_) is weaned to 0.25 while targeting a transcutaneous oxygen saturation of 86–94 % (opening pressure or CDP_O_). The CDP is then stepwise decreased until oxygenation deteriorates, indicating alveolar/saccular collapse (closing pressure or CDP_C_). Following a brief increase of CDP_O_, the pressure is then set at 2 cm H_2_O above the closing pressure (optimal pressure or CDP_OPT_). The pressure amplitude is adjusted to maintain the transcutaneous partial carbon dioxide pressure between 5.3 and 8.0 kPa.

Next, the position of the endotracheal tube is checked on a CXR and patients are treated with 100–200 mg/kg exogenous surfactant (Curosurf, Chiesi Pharmaceutici, Parma, Italy). The following surfactant treatment CDP_OPT_ is once more determined as described above.

Finally, umbilical lines are placed in infants with a gestational age below 30 weeks, a birth weight below 1000 g, or ongoing cardiorespiratory instability. In these infants, the position of the umbilical lines is checked with a second CXR.

Patients were prospectively included in the study if they were ventilated with open-lung HFV for RDS and had a CXR taken within a predefined time frame of 2 h after reaching the presurfactant CDP_OPT_. Although not the primary focus of this study, we also performed postsurfactant analysis if a second CXR was taken within 6 h after reaching postsurfactant CDP_OPT_.

### Data collection and analysis

Bedside digital anteroposterior CXRs were taken with the infant placed in supine position. The date and time of the different stages of lung recruitment before and after surfactant were prospectively collected and linked to the date and time of the CXR. The anonymized CXRs were interpreted, in random order, by two pediatric radiologists blinded from all information related to the patients.

Lung volume was assessed by scoring radiolucency, lung inflation, and the presence of air leaks. Radiolucency was graded as follows: grade 0: normal radiolucent lung fields with sharp cardiac and diaphragmatic margins; grade 1: slightly reduced radiolucency with still sharp cardiac and diaphragmatic margins; grade 2: markedly reduced radiolucency with retained cardiac and diaphragmatic margins; grade 3: severely reduced radiolucency with air bronchogram and blurred cardiac and diaphragmatic margins; and grade 4: almost completely white lung fields with or without air bronchogram and barely visible cardiac and diaphragmatic margin [10, 21].

Lung hyperinflation was defined by the presence of an inverted diaphragm shape and/or a diaphragm position visible at more than nine posterior ribs and/or lung bulging. Hyperinflation was graded mild, moderate, or severe, correlating with, respectively, 1, 2, or 3 of the aforementioned signs of hyperinflation.

Air leaks were classified as pneumothorax, tension pneumothorax, pulmonary interstitial emphysema, pneumomediastinum, pneumopericardium, and/or subcutaneous emphysema. Pulmonary interstitial emphysema was defined as either linear opacities with a diameter of 1–2 mm or cystic opacities (>2 mm) with a heterogeneous distribution across (part of) the lungs or large bullous opacities not based in congenital anomalies or extrapleural air.

The left and right lungs were scored separately, and in the case of differences between both lungs, the highest score was used for analysis. In case of disagreement, both radiologists reevaluated the CXRs in an attempt to reach consensus.

In addition to the CXR, the following characteristics were retrieved from the patient charts: gestational age, birth weight, use of antenatal steroids, route of delivery, 5-min Apgar score, gender, singleton birth, surfactant therapy (yes/no), and the presence of air leaks during the admission. Data on ventilation settings, including airway pressures and FIO_2_, at the different stages of lung recruitment, were prospectively recorded for each patient.

Statistical analysis was performed using SPSS version 18 (SPSS, Chicago, IL). Data are presented as mean ± standard deviation (SD) or median and interquartile range (IQR) depending on their distribution. The difference in radiolucency and inflation before and after surfactant was analyzed by the marginal homogeneity test.

## Results

Between March 2004 and August 2005, a total of 103 consecutive patients were treated with open-lung HFV and 69 of these infants were included in the study (Table 1). Reasons for exclusion were as follows: no CXR taken within the predefined time frame (*n* = 20), poor CXR quality making meaningful assessment of lung condition not possible (*n* = 11), and not able to retrieve the CXR from the archive due to corrupt data (*n* = 3). There were no significant differences between included and excluded infants in terms of death or signs of barotrauma during the entire admission.

**Table 1:** Patient and clinical characteristics

	*N* = 69
Gestational age (weeks)	29 ± 2.4
Birth weight (g)	1250 ± 474
<1000 g, no. (%)	24 (33)
Antenatal steroid, no. (%)	54 (74)
Caesarean Section, no. (%)	32 (44)
Male gender, no. (%)	45 (62)
Singleton, no. (%)	61 (84)
5-min Apgar score, median [IQR]	8 [7, 9]
Air leaks, no. (%)^b^	4 (6)
Surfactant, no. (%)	67 (97)
CDP at first CXR, cmH_2_O	15 ± 4.7
CDP at second CXR, cmH_2_O^a^	9 ± 2.4
FIO_2_ at first CXR, median [IQR]	0.25 [0.21, 0.28]
FIO_2_ at second CXR median [IQR]^a^	0.21 [0.21, 0.25]

Data expresses as mean ± SD, unless stated differently

*CDP* continuous distending pressure, *CXR* chest X-ray, *FIO*_*2*_ fraction of inspired oxygen

^a^ *n* = 39

^b^All air leaks were diagnosed during the entire admission

The first CXR was made at a median [IQR] of 3.5 h [2, 9.5] with a minimum of 0.3 h and a maximum of 11 h after birth and 13 min [4, 32] after completing the presurfactant recruitment procedure. The surfactant was administered in 67 (97 %) of the included patients, but in only 39 patients, a second CXR was made at a median [IQR] of 135 min [82, 201] after completing the postsurfactant recruitment procedure. In all 39 infants, the reason for this second CXR was placement of umbilical lines. In 11 (10 %) of the CXRs, scoring differed between the radiologists and the final result was therefore based on consensus.

The presurfactant recruitment procedure resulted in a FIO_2_ ≤0.25 in 49 (71 %) and ≤0.30 in 63 (91 %) patients. Assessment of the radiolucency showed a median [IQR] score of 2 [1, 3] with the majority of infants (64 %) having a score ≤2. Lung recruitment resulted in only mild and moderate signs of hyperinflation in, respectively, eight (12 %) and five (7 %) patients. None of the CXRs showed signs of air leaks (Table 2).

**Table 2:** Frequency distribution radiolucency, hyperinflation, and air leaks

	Before surfactant *N* = 69	After surfactant *N* = 39
Lung radiolucency, no. (%)		
Grade 0	5 (7)	7 (18)
Grade 1	22 (32)	20 (51)
Grade 2	17 (25)	8 (21)
Grade 3	23 (33)	3 (7)
Grade 4	2 (2)	1 (3)
Median grade [IQR]	2 [1, 3]	1 [1, 2]
Lung hyperinflation, no. (%)		
Mild	8 (12)	8 (21)
Moderate	5 (7)	1 (3)
Air leak, no. (%)	0	0

Following surfactant treatment, the median FIO_2_ was 0.21 (Table 1) and there was a clear shift to lower radiolucency scores, with a median [IQR] score of 1 [1, 2] (Table 2). On an individual level, the radiolucency score improved in 62 % of the 39 paired CXRs (*p* < 0.001, Table 3). After surfactant therapy, there was no change in the hyperinflation score in 23 (59 %) patients; in 7 (18 %) patients, the score increased to mild or moderate; and in the remaining 9 (23 %) patients, the signs of hyperinflation decreased or disappeared. Again, air leaks were not encountered.

**Table 3:** Change in radiolucency score before and after surfactant administration

Grade difference^a^	No. (%)	
−2	1 (2.6)	Same or worse 15 (38 %)
−1	4 (10.3)	
0	10 (25.6)	
1	14 (35.9)	Improved 24 (62 %)
2	8 (20.5)	
3	2 (5.1)	

^a^Obtained by subtracting radiolucency grade before and after surfactant therapy

A total of nine infants (13 %) had an intraventricular hemorrhage grade 3 or 4 and no cases of cystic periventricular leukomalacia were found.

## Discussion

To our knowledge, this is the first study that describes the effect of an individualized oxygenation-guided lung recruitment procedure during HFV on the CXR characteristics of preterm infants with RDS. Our study, which included all patients receiving open-lung HFV in a prospective manner, shows that hyperinflation is uncommon and the risk of air leaks is low when using this individualized approach.

Since its introduction in the early 1990s, the high mean airway pressures applied during HFV have been associated with an increased risk of air leaks [3, 4, 19]. Despite the fact that we used a relatively low FIO_2_ (0.25) to define an open lung, thereby subjecting the infants to relatively high airway pressures to recruit the lung, the CXR only showed mild to moderate signs of hyperinflation in a small proportion of infants. Consistent with this finding, no air leaks were seen during or immediately after the recruitment procedure.

These findings are probably best explained by the fact that the opening pressures to recruit the lung were tailored to the individual severity of RDS. In addition, airway pressures were rapidly reduced once the lung was recruited, taking advantage of the lung hysteresis that is present in preterm infants with RDS, ensuring that lung volume is maintained at the lowest possible airway pressures [13].

Despite the fact that exogenous surfactant will increase lung volume and improve its stability within minutes after administration [14], surfactant treatment did not increase the risk of hyperinflation or air leaks. Again, this finding is probably best explained by the fact that airway pressures were rapidly weaned after surfactant treatment, aiming to find the new optimal distending pressure to stabilize the lung.

To allow for a better comparison with previous studies on HFV, we also assessed the number of air leaks during the entire admission and found a relatively low incidence of 6 % [17, 20]. These results suggest that the low risk of lung hyperinflation and air leaks in the first hours of ventilation is sustained over a longer period over HFV. The fact that the included infants were slightly more mature than those reported in other HFV trials may also have contributed to the relatively low incidence of air leaks in our study.

The radiolucency score following lung recruitment is considerably lower than those previously reported in conventionally ventilated preterm infants with RDS [2, 7, 8, 23]. There may be several reasons explaining this finding.

First and probably most important, the recruitment procedure used in our study resulted in opening and thus better aeration of collapsed alveoli/sacculi, which improved not only oxygenation (low FIO_2_) but also radiolucency of the CXR. Such a recruitment procedure was not applied in previous studies including conventionally ventilated patients.

Second and in contrast to our study, infants in most of the previous studies did not receive antenatal steroids, which are known to reduce the severity of RDS [16]. However, the fact that a more recent study in conventionally ventilated preterm infants with RDS also showed higher radiolucency scores makes this a less likely explanation for the observed difference in lung aeration [7].

Finally, other differences in patient characteristics between studies, such as gestational age, birth weight, and timing of the CXR, may also have affected the radiolucency in RDS. However, most of these differences were small and the impact on radiolucency score was probably limited as indicated by the comparable FIO_2_ at the start of ventilation in this and in other studies.

Surfactant treatment resulted in a significant improvement in the radiolucency score, a finding consistent with previous studies in conventionally ventilated preterm infants with RDS [2, 7, 8, 23]. Based on the median radiolucency scores, this improvement is modest, a finding best explained by the fact that the lung was already recruited at the time of surfactant treatment. It is, however, important to realize that the postsurfactant radiolucency scores were obtained at much lower airway pressures than before surfactant treatment. This finding once again confirms the surface tension-lowering properties of surfactant, allowing stabilization of lung volume at much lower airway pressures compared to the surfactant-deficient lung [14].

To date, only one study compared CXR characteristics in infants with RDS ventilated with either HFV or conventional ventilation [11]. Helbich and colleagues reported a median RDS severity score of 4 on the first day of life, independent of the ventilation mode and after surfactant treatment. This radiolucency score in HFV infants is much higher than the median score of 1 in our study. This discrepancy is probably best explained by the fact that Helbich and colleagues used the CXR (inflation up to the dorsal part of the ninth rib) to monitor lung volume optimization and not oxygenation. A previous study showed that the correlation between lung inflation on the CXR and the functional residual capacity is probably poor [21]. This line of reasoning is also supported by the large differences in median FIO_2_ after lung recruitment and surfactant in our study (0.21) and the study of Helbich and colleagues (0.54).

This study has several limitations that need to be addressed.

First, the timing of the CXR after lung recruitment and surfactant varied between patients, which may have caused some variation in the CXR characteristics.

Second, the fact that CXRs were only available in approximately half of the patients may have biased the results. However, it is unethical to perform CXRs for research purposes only in infants not treated with surfactant. Furthermore, the decision to perform a CXR after surfactant was not based on the response to lung recruitment but solely on the need to insert umbilical lines, thereby limiting the level of bias. The fact that we found an improvement in radiolucency score similar to previous studies in conventionally ventilated patients seems reassuring.

Third, although most commonly used by clinicians, CXR is not the best tool to assess lung volume in preterm infants. Results may be different when using other monitoring tools.

Fourth, the radiolucency score after surfactant treatment may have been impacted by the administered dose. Unfortunately, this information could not be reliably retrieved from the patient charts, thus making it impossible to explore this with subgroup analysis. Finally, more modern high-frequency ventilators allow for accurate tidal volume monitoring and this may further enhance the safety of an open-lung ventilation strategy.

In conclusion, this study shows that hyperinflation is uncommon and the risk of air leaks is low when using an individualized oxygenation-guided recruitment procedure during HFV in relatively mature preterm infants with RDS. Lung recruitment during HFV results in a relatively low radiolucency score compared with previous studies using conventional mechanical ventilation. These results indicate that from a lung injury perspective, open-lung HFV is relatively safe in preterm infants with RDS.

## Abbreviations

BPD: Bronchopulmonary dysplasia
CDP: Continuous distending pressure
CDP_C_: Closing continuous distending pressure
CDP_O_: Opening continuous distending pressure
CDP_OPT_: Optimal continuous distending pressure
CXR: Chest X-ray
FIO_2_: Fraction of inspired oxygen
HFV: High-frequency ventilation
IQR: Interquartile range
nCPAP: Nasal continuous positive airway pressure
RDS: Respiratory distress syndrome
SD: Standard deviation
